# Scanning Magnetic Microscopy Using a High-Sensitivity Room-Temperature Tunnel Magnetoresistance Sensor for Geological Applications

**DOI:** 10.3390/s26031075

**Published:** 2026-02-06

**Authors:** Hirokuni Oda, Kosuke Fujiwara, Naoto Fukuyo, Hitoshi Kubota, Tomohiro Ichinose, Mikihiko Oogane, Seiji Kumagai, Hitoshi Matsuzaki, Taizo Uchida, Miki Kawabata, Jun Kawai

**Affiliations:** 1 Research Institute of Geology and Geoinformation, Geological Survey of Japan (GSJ), National Institute of Advanced Industrial Science and Technology (AIST), Central 7, 1-1-1 Higashi, Tsukuba 305-8567, Ibaraki, Japan; 2 Spin Sensing Factory Corp., Sendai 980-0845, Miyagi, Japan; kosuke.fujiwara@spintronics.co.jp (K.F.); seiji.kumagai@spintronics.co.jp (S.K.); h.matsuzaki@spintronics.co.jp (H.M.); 3 Department of Geography, Faculty of Letters, Hosei University, 2-17-1 Fujimi, Chiyoda 102-8160, Tokyo, Japan; naoto.fukuyo.53@hosei.ac.jp; 4 Research Institute for Hybrid Functional Integration, National Institute of Advanced Industrial Science and Technology (AIST), Central 2, 1-1-1 Umezono, Tsukuba 305-8568, Ibaraki, Japan; hit-kubota@aist.go.jp (H.K.); tomohiro.ichinose@aist.go.jp (T.I.); 5 Department of Applied Physics, Graduate School of Engineering, Tohoku University, Sendai 980-8579, Miyagi, Japan; mikihiko.ogane.e4@tohoku.ac.jp; 6 Applied Science Program, Graduate School of Integrated Arts and Sciences, Kochi University, 2-5-1 Akebonocho, Kochi 780-8520, Kochi, Japan; b24d6a05@s.kochi-u.ac.jp; 7 Applied Electronics Laboratory, Kanazawa Institute of Technology, Kanazawa 920-1331, Ishikawa, Japan; mkawabat@neptune.kanazawa-it.ac.jp (M.K.); j-kawai@neptune.kanazawa-it.ac.jp (J.K.)

**Keywords:** scanning magnetic microscopy, tunnel magnetoresistance (TMR) sensor, superconducting quantum interference device (SQUID), geological thin section, magnetic point source, calibration, frequency response, sensor characteristics

## Abstract

This paper reports magnetic microscopy using high-sensitivity room-temperature tunnel magnetoresistance (TMR) devices for thin geological sections. The sensitivity region of the TMR sensor has dimensions of 178 µm (L) × 0.1 µm (W) × 100 µm (H), consisting of two TMR devices. Magnetic images were obtained for a vertically magnetized Hawaii basalt thin section in two sensor configurations, with the sensor length aligned parallel to the X- (lift-off = 174 μm) and Y-axes (lift-off = 200 μm), without introducing anisotropic distortion in the magnetic images. Although the magnetic images obtained with a scanning SQUID microscope (SSM) were similar, slight discrepancies were observed in the high-spatial-resolution region. A magnetic point source (50 μm × 50 μm) with a perpendicular magnetization film was prepared for evaluation. The SSM measurements showed a clear magnetic dipole at an angle of approximately 1° from the vertical direction. The FWHMs for both the SSM and TMR sensors increased linearly with lift-off. However, the peak magnetic fields, magnetic moments, and dipole tilts of the TMR sensor were significantly larger than those of the SSM sensor. This discrepancy may be due to the vertical extent of the active region of the TMR sensor, as well as due to sensor noise and drift.

## 1. Introduction

Scanning magnetic microscopy (SMM) is a technique for mapping magnetic fields of samples with high spatial resolution and sensitivity. It is used in the scientific, technological, and medical fields to image magnetization and current source distributions. SMM for geological applications has developed over the past twenty years, which entails interpreting paleomagnetic and rock magnetic manifestations at macroscopic scales by visualizing distributions of submillimeter- to submicrometer-scale magnetizations. Previous studies have extensively discussed submillimeter-scale magnetostratigraphy of marine ferromanganese crusts [1], meteorites [2,3], volcanic rocks [4], and magnetic moment measurements of single zircon crystals for paleointensity estimates [5,6]. SMM has evolved using a variety of magnetic sensors such as the magneto-impedance (MI) device [7], Hall-effect device [8], superconducting quantum interference device (SQUID) [1,4,9,10,11,12], including high-Tc SQUID [13], and TMR device [14,15,16]). Quantum diamond devices [3,5,17,18]) are an emerging technology facilitating image capture of the area without electromechanical scanning. Magneto-optical microscopy provides another approach for visualizing magnetic domains and related phenomena [19]. Furthermore, submillimeter-scale magnetic scanning has been demonstrated using an optically pumped magnetometer [20].

Among these, SQUID sensors exhibit the best sensitivity, better than 10 fT at 1 Hz, using a superconducting pickup coil with a diameter of 45 mm [21]. Furthermore, SQUID sensors can act as magnetic microscopes. The scanning SQUID microscope (SSM) at the Geological Survey of Japan (GSJ), National Institute of Advanced Industrial Science and Technology (AIST), has a magnetic field noise level of 1.1 pT/Hz at 1 Hz and a pickup loop measuring 200 μm × 200 μm [12]. However, SQUID sensors are limited by the need to be cooled to cryogenic temperatures, which is costly and labor-intensive. In addition, the lift-off for a room-temperature SQUID microscope is typically greater than 80–100 μm, which limits the sensitivity and the resolution in the scanning mode [14]. Although SQUID electronics with flux counting can extend dynamic range (e.g., 2G Enterprises superconducting rock magnetometer), the upper limit remains below Earth’s field. The SSM at GSJ measures fields up to ±7000 nT [11].

Tunnel magnetoresistance (TMR) devices are prospective for magnetic microscopy applications because of their extended dynamic range (picotesla to millitesla), wideband frequency response, small active area, ease of measurement, and cost efficiency. In TMR, electrons tunnel through a thin insulating layer between two ferromagnetic layers under a bias voltage across magnetic tunnel junctions (MTJ). One layer has a fixed magnetic moment, while the other responds to the applied field. The tunneling current depends on the relative magnetization angle, making resistance a function of the applied magnetic field. The lower bound of the dynamic range of a TMR-based magnetic microscope can be extended to the sub-nanotesla levels. Recent advances of TMR sensors with CoFeB/MgO/CoFeB-MTJs have improved the sensitivity, allowing the detection of weak biomagnetic fields, such as brain magnetic fields [22]. We previously demonstrated magnetic microscopy using high-sensitivity room-temperature TMR sensors developed for magnetoencephalography (MEG), in combination with an XYZ stage developed for SSM [23]. TMR sensors used are composed of serially connected TMR elements with a total length of 2684 μm.

While SQUID-based SMM provides high sensitivity, the need for cryogenic operation limits its usability. In contrast, TMR sensors operate at room temperature and offer a wide dynamic range with a small active area. Our previous study showed that TMR sensors could yield viable magnetic images of geological thin sections with fields of several hundred nanotesla, although the elongated active area introduced image anisotropy. In this study, we present a 178 μm-long TMR sensor, which is shorter than our previous designs and is intended to achieve more isotropic magnetic images. Magnetic images were obtained for a Hawaii basalt thin section and a magnetic point source, both magnetized vertically. Image distortion was evaluated together with the frequency characteristics to compare with those of the previous TMR sensors and to estimate the magnetic moment sensitivity. In addition, the magnetic images were compared with those obtained using scanning SQUID microscopy.

## 2. Materials and Methods

### 2.1. Experimental Setup and Specifications of the TMR Sensor

**Figure 1:**
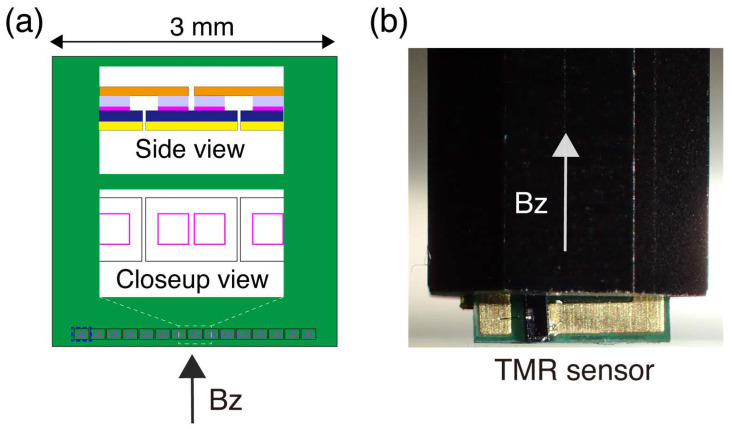
TMR sensor used for the study. (**a**) Details of the original TMR sensor before length reduction. The green area represents a silicon chip with a series of paired TMR elements (an island) aligned along the upper edge. Fifteen islands are connected serially. Insets are close-up and side views of an island. Each island shares an electrode (yellow) and a free layer (dark blue; 100 μm × 178 μm). The pink color indicates MTJs, and the light blue represents pinned layers. The gaps between islands are 1 μm. Each island is connected to the adjacent islands via the upper electrode (orange). For this study, one island (the leftmost one, enclosed by thick blue dashed lines) is used as a sensor, which includes two TMR devices. The thickness of a free layer is 0.1 μm, which corresponds to the width of the magnetic field sensing region. (**b**) Appearance of the TMR sensor. The black part is an aluminum body holding the TMR sensor, which is tightly fixed to a vertically oriented aluminum frame in the magnetic shield case.

A new TMR sensor is prepared for the measurements (Figure 1) utilizing that for MEG with a length of 2684 μm [24], which is similar to but is different from the previous demonstrations [23]. The original TMR sensor (Spin Sensing Factory Corp., Sendai, Japan) is composed of multiple serially connected TMR elements and a flux concentrator, allowing high magnetic field detectivity (Figure 1a [19]). The flux concentrator is removed to achieve a high spatial resolution, which is appropriate for magnetic microscopy. To achieve high spatial resolution, the length of the TMR sensor was reduced to 178 μm by reducing the number of serially connected TMR elements to one, which comprises a pair of TMR devices.

To demonstrate and evaluate magnetic microscopy with TMR sensors, an XYZ stage and controller originally developed for the SSM at GSJ, AIST [11], were utilized. The experimental setup was similar to that reported previously [23]. TMR sensors were installed on a two-layer magnetic shield above the sample holder. Each sample was mounted on the holder, and the vertical magnetic field component was scanned along X and Y directions at a lift-off of approximately 300 μm. Scanning was carried out using SQUIDMagScan software Version 2.02b [14,25], which enabled data collection along +Y lines with a stepwise movement in the +X direction. For signal acquisition, the TMR sensor was connected to the input of the XYZ stage controller via a DC preamplifier based on a Wheatstone bridge circuit and a DC amplifier, as in [23]. A precision DC power supply (E3620A, Agilent Technologies, Santa Clara, CA, USA) supplied 10 V to the DC amplifier with a fixed gain of 100 dB.

### 2.2. Sensor Calibration and Noise Characterization

Calibration of the TMR sensor sensitivity was performed by applying a 10 mA line current generated by a precision current source [11,23]. The sensitivity of the sensor was estimated by fitting the measured magnetic field values to the theoretical curve of an infinite line current. Based on this fitting, the calibration factor was determined to be 700 nT/V (Appendix A). Blank measurements were conducted using the TMR sensor without moving the XYZ stage at a sampling frequency of 50 Hz using SQUIDMagScan Version 2.02b and an XYZ stage controller, similar to a previous study [23]. To evaluate noise, the dataset was divided into multiple segments, and a periodogram was produced by stacking the spectral densities after applying the Hanning window using Igor Pro™ software Version 9.05 (Build 56551).

### 2.3. Sample Specifications, Experimental Methods, and Data Processing

Hawaiian basalt was used as an example of a geological thin section previously studied using SSM [11] and TMR sensors [23]. The thin section was scanned using a flatbed optical color scanner (GT-X980, Seiko Epson Corp., Tokyo, Japan) at 4 × 4 μm pixel resolution to obtain an optical image. Anhysteretic remanent magnetization (ARM) was applied in a DC magnetic field of 50 μT combined with an AC field of 80 mT, oriented downward and perpendicular to the thin section [11,23]. In addition, a magnetic point source composed of a perpendicular magnetization film was prepared for measurement on a silicon substrate at AIST. The perpendicularly magnetized film consisted of a thin CoFe layer sandwiched between two MgO layers [26]. The detailed stacking of the layers was as follows: Ta(5)/TaB(5)/MgO(2.3)/Ir(0.15)/CoFe(0.8)/MgO(1)/capping layers (nanometer thickness). Capping layers were deposited to prevent degradation of the magnetic properties. The CoFe layer was deposited at 100 K using a sputtering system (EXIM; Tokyo Electron, Ltd., Tokyo, Japan). Cryogenic temperature deposition effectively suppressed the roughness of the CoFe film. An ultrathin Ir layer was inserted into the multilayer to enhance the perpendicular magnetic anisotropy of CoFe. After film deposition, the multilayer was annealed at 400 °C in the Earth’s magnetic field without a strong applied field. This resulted in downward perpendicular magnetization with respect to the film surface deposited on the silicon substrate. Subsequently, a 50 μm × 50 μm square pattern is fabricated in the perpendicular magnetization film using a resist, and the film is milled in the chamber for 24 min at 200 V, 60 mA. Finally, the pattern was covered with SiO_2_ via sputtering in the chamber, and the excess margin was trimmed using a diamond wire saw at GSJ-Lab.

For a geological thin section, magnetic field scanning was performed using a 100 μm rectangular grid. The TMR sensor was used for magnetic scans in the y- and x-directions. The XYZ stage was moved along the Y and X axes at a speed of 50 mm/min. The stepping motor was stopped, and then data acquisition at each measurement point was performed after 0.3 s. Raw magnetic field data were corrected for drift as described in [11,23,25]. Measurements at the upper and lower margins of the thin section were taken and treated as zero magnetic field. After drift correction, a median filter was applied to the selected images to remove noise spikes.

The magnetic point source was measured using the TMR sensor at 25 μm grid spacing in the previous configurations. For each axis, a pair of measurements was performed, and the average magnetic image was used for analysis. The magnetic point source was also measured with SSM at 50 μm grid spacing and compared with the results of the TMR measurements. A magnetic dipole was fitted to the magnetic images of the magnetic point source using the Igor Pro nonlinear regression fitting algorithm implemented in ProcSQMicro Version 20250212 [25]. Seven independent parameters were fitted: the dipole moment (*M_x_*, *M_y_*, *M_z_*), dipole position (*P_x_*, *P_y_*, *P_z_*), and offset (a constant magnetic field value for the background).

Upward continuation is the process of transforming the potential field data (magnetic and gravity) from a flat observation plane to a higher plane [27]. An upward continuation filter was applied in this study to compare the magnetic images measured with different lift-off values. The filter was applied in 2-D frequency space using an FFT with a function implemented in ProcSQMicro Version 20250212 [23,25].

## 3. Results

### 3.1. Noise Characteristics of the TMR Sensor

**Figure 2:**
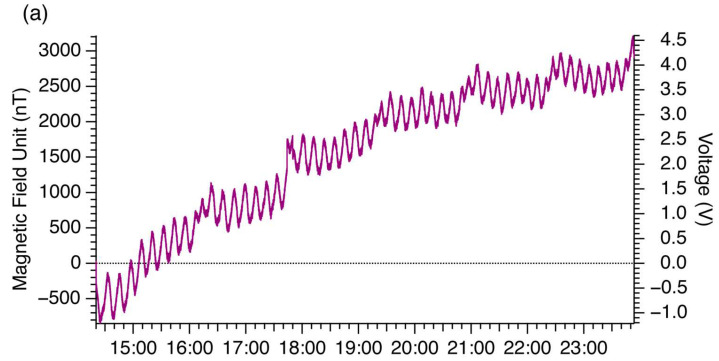

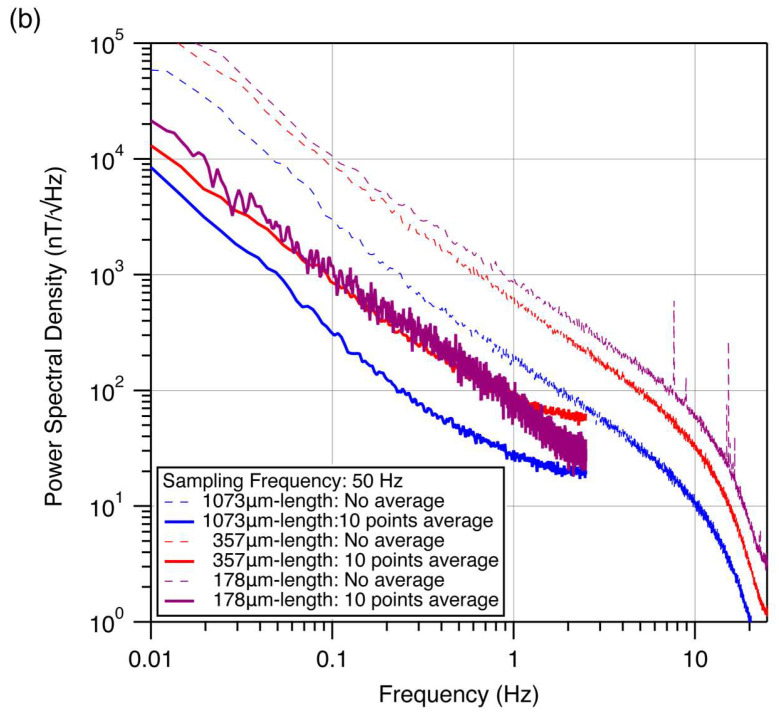
Background magnetic field and spectrum of TMR sensors. (**a**) Temporal variation of measured magnetic field (**left**) and voltage (**right**) for the new TMR sensor of length 178 μm used in this study. (**b**) Power spectral density of the magnetic field for the new TMR sensor (purple), which is compared with TMR sensors of lengths 1073 μm (blue) and 357 μm (red). Dashed and solid lines represent measurements with and without the 10-point average, respectively. Curves for TMR sensors of lengths 1073 μm and 357 μm are reproduced from Figure 2b of [23].

Figure 2 shows the background magnetic field. The purple line in Figure 2a represents the temporal change in the magnetic field signals (left axis) converted from the measured voltage (right axis) using the calibration constants. Characteristic fluctuations with a period of approximately 12 min were evident, as well as those with a period of ~1.3 h, both of which are nearly synchronous with the temperature variations that may originate from the switching cycle of the air conditioner. In fact, TMR sensors are sensitive to temperature changes [28,29]; however, our drift-correction procedure effectively compensates for fluctuations with characteristic times longer than ~5 min. The power spectral density (PSD) of the background measurements (Figure 2b) exhibits patterns typical of 1/f noise. PSD of the raw data for the new TMR sensor (Sensor #3; purple line; 178 μm length) was larger than that of Sensor #2 (357 μm length; dashed red line). Sensor #3 exhibited 10,950, 840, and 60 nT/√Hz at 0.1, 1, and 10 Hz, respectively, whereas Sensor #2 exhibited 8790, 560, and 33 nT/√Hz at 0.1, 1, and 10 Hz, respectively. The corresponding PSD ratios are 1.26, 1.49 and 1.82 for 0.1, 1, and 10 Hz, averaging at about 1.5. This is comparable to the square root of the ratio of the sensor lengths (√357/178 = 1.41). In addition, the PSD was calculated for the same dataset using a running average of 10 points, which was also larger than that of Sensor #2 but closer to it.

### 3.2. Magnetic Images of a Geological Thin Section

**Figure 3:**
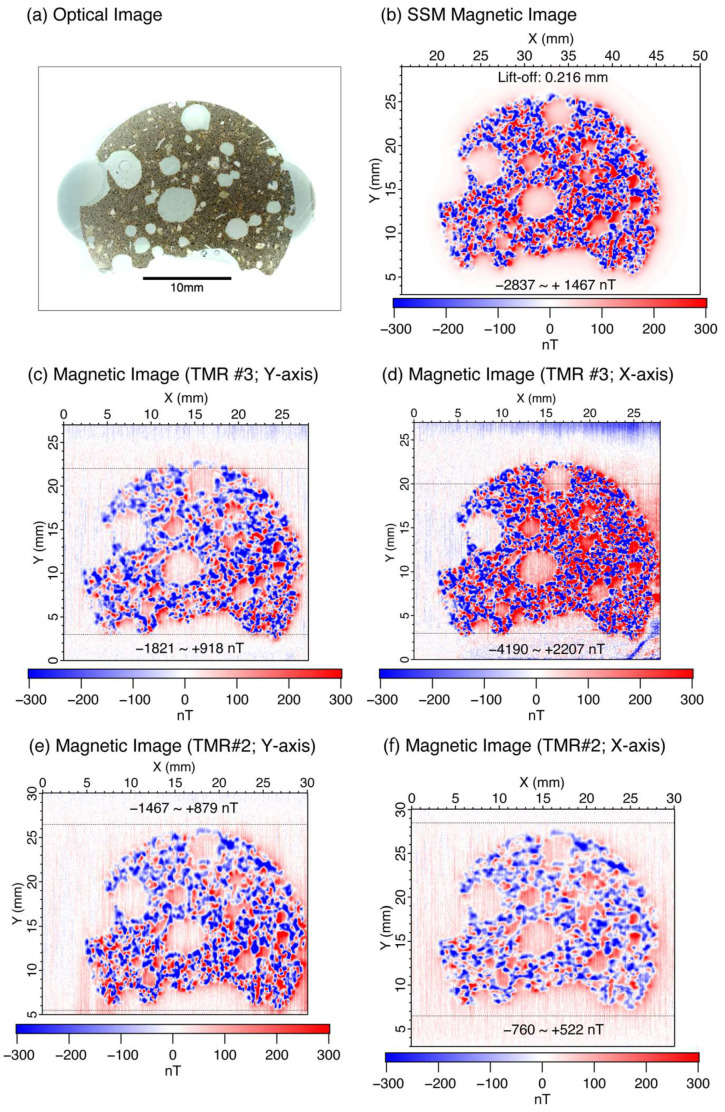
Magnetic images of basalt thin section using TMR sensors. (**a**) Optical image acquired using an optical scanner. (**b**) Magnetic image measured using a scanning SQUID magnetic microscope (SSM). (**c**,**d**) Magnetic images obtained using the new 178-μm-long TMR sensor with the length direction parallel to y- and x-axes. Each grid point is a 10-point average of measurements with a sampling rate of 50 Hz. (**e**,**f**) Magnetic images obtained using a 357-μm-long TMR sensor with the length direction parallel to y- and x-axes. Magnetic images use a color scale from −300 nT (blue) to +300 nT (red). Values below −300 nT and above −300 nT are clipped to the corresponding end colors. The actual minimum and maximum magnetic field values are indicated in each subfigure. Figure 3a, Figure 3b, Figure 3e, and Figure 3f are reproduced from Ref [23], Figure 3a, Figure 4b, Figure 5e, and Figure 5g, respectively. The horizontal dotted lines mark the boundaries of the regions used for drift correction, with the outer portions of the image employed for determining the drift.

Figure 3 shows magnetic field images of the basalt thin-section sample acquired with the ARM in a downward direction, together with an optical image (Figure 3a). Figure 3b shows a magnetic image measured using the SSM after drift correction [11]. The lift-off was estimated to be 216 μm for minimum and maximum magnetic fields of −2837 nT and 1467 nT, respectively. Figure 3c,d show the magnetic images measured using TMR Sensor #3, with the longitudinal direction oriented parallel to the y- and x-axes, respectively. The magnetic field value ranges from −1821 to 918 nT and −4190 to 2207 nT. Based on the minimum and maximum magnetic field values and color images, the magnetic image of Figure 3c is measured with a larger lift-off than that of Figure 3b for the SSM magnetic image. Similarly, the magnetic image of Figure 3d is measured with a smaller lift-off than that of Figure 3b for the SSM magnetic image. In Figure 3c,d, there is no visible distortion related to the elongation of TMR Sensor #3. In contrast, the magnetic images in Figure 3e,f measured with TMR Sensor #2 (length of 357 μm [23]) exhibit a slight stretching distortion of the image along the y-and x-axes, respectively.

### 3.3. Magnetic Images of a Magnetic Point Source

**Figure 4:**
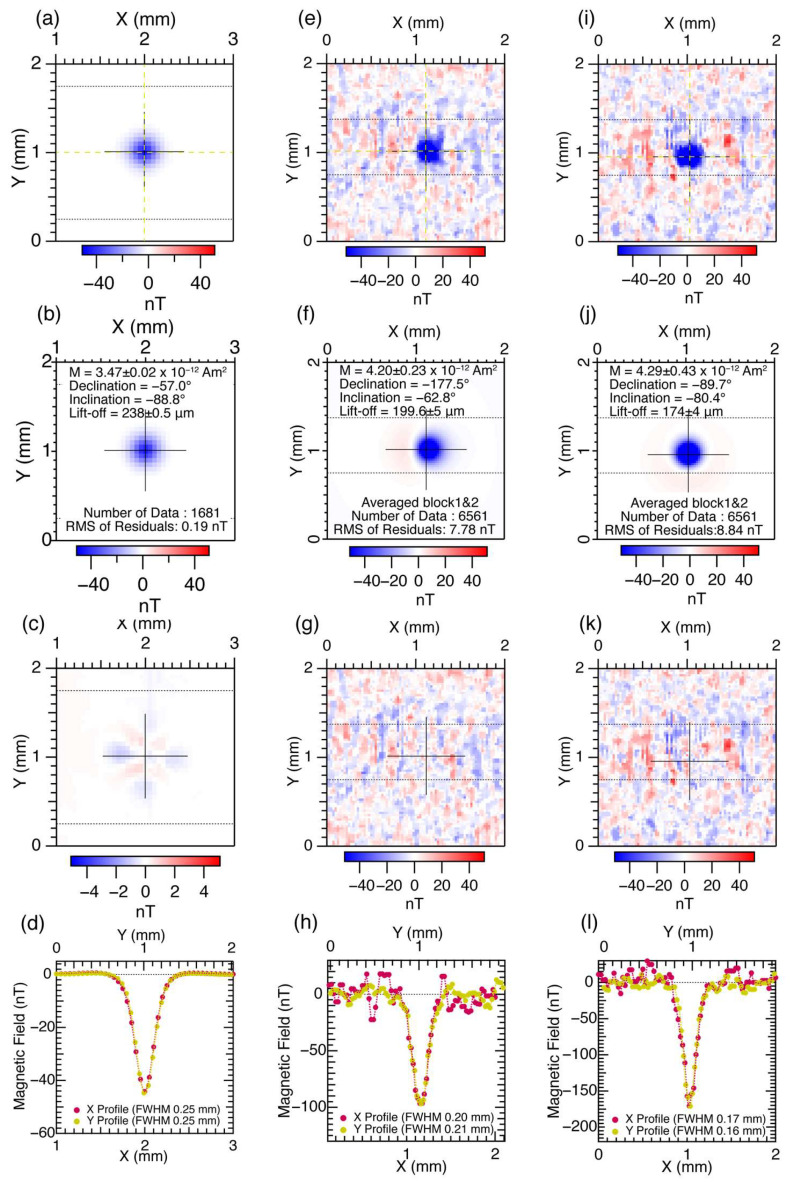
Magnetic images of a point source using SSM and TMR sensor. Red and blue colors correspond to positive and negative magnetic field values, respectively. The point source is a multilayered perpendicularly magnetized CoFe film (see text for details of the film). The magnetic point source exhibits downward magnetization and has a square shape with dimensions of 50 μm × 50 μm. (**a**) Magnetic field image of the point source measured with the SSM. (**b**) Magnetic image of a dipole fitted to the magnetic field values in Figure 4a. (**c**) Residual magnetic field values for the fitted dipole in Figure 4b. (**d**) Horizontal and vertical profiles of the magnetic field image in Figure 4a along the X-axis (purple circles) and the Y-axis (orange circles). The dotted lines represent curves based on cubic spline interpolation, which are used to estimate FWHM values. (**e**,**i**) Magnetic field images of the point source measured with the TMR sensor with the longitudinal direction along the Y- and X-axes. (**f**,**j**) Magnetic images of a dipole fitted to the magnetic field values in Figure 4e,i. (**g**,**k**) Residual magnetic field values for fitted dipole in Figure 4f,j. (**h**,**l**) Horizontal and vertical profiles of magnetic field image in Figure 4e,i along X- and Y-axes. Dotted lines represent curves based on smoothing spline interpolation, which are used to estimate FWHM values. Thin black crosses in the magnetic images indicate the positions of the best-fit dipoles. The horizontal dotted lines in Figure 4a,c,e,g,i,k mark the boundaries of the regions used for drift correction, with the outer portions of the image employed for determining the drift.

Figure 4 shows magnetic field images of the point source with vertical magnetization acquired with TMR Sensor #3 and the SSM. Figure 4a shows a magnetic field image of the point source measured with the SSM on 50 μm grids. From the symmetrical image in blue, it is evident that the point source exhibits a downward-pointing magnetization. A magnetic dipole is fitted to the magnetic image (Figure 4b), which results in a magnetic moment of 3.47 ± 0.02 × 10^−12^ Am^2^, declination (counter-clockwise from X-axis) of −57.0°, and inclination (angle from the horizontal surface; positive value corresponds to upward direction) of −88.8°. The lift-off estimated as part of the regression is 238 ± 0.5 μm, which is slightly larger than that estimated utilizing a precision line current scan (222 μm). Figure 4c shows the residual magnetic-field values after fitting with the magnetic dipole. The root-mean-square of the residuals over the entire 1681 data points is 0.19 nT, which is relatively small.

Figure 4e shows a magnetic-field image of the point source measured using the TMR sensor in the longitudinal direction aligned parallel to the Y-axis. Two sets of magnetic images were obtained and averaged for each pixel after the drift correction. After averaging, a 3 × 3 median filter was applied to reduce noise. Similar to the SSM magnetic image analyses, a magnetic dipole was fitted to the magnetic image (Figure 4f), resulting in a magnetic moment of 4.20 ± 0.23 × 10^−12^ Am^2^, a declination of −177.5°, and an inclination of −62.8°. The lift-off estimated from the regression was 200 ± 5 μm, smaller than that for the SSM. The residual magnetic field values after fitting with the magnetic dipole are shown in Figure 4g. The root mean square of the residuals for the entire 6561 pixels was 7.78 nT, which was much larger than that for the SSM.

Figure 4i shows a magnetic field image of the point source measured using the TMR sensor in the longitudinal direction aligned parallel to the X-axis. A magnetic dipole is fitted to the magnetic image (Figure 4f), which results in a magnetic moment of 4.29 ± 0.43 × 10^−12^ Am^2^, declination of −89.7°, and inclination of −80.4°. The lift-off estimated as part of the regression was 174 ± 4 μm, which was smaller than that for the longitudinal direction aligned parallel to the Y-axis. Figure 4j shows the fitting residuals. The root mean square of the residuals for the entire 6561 pixels was 8.84 nT, which was similar to that for the longitudinal direction aligned parallel to the Y-axis.

## 4. Discussion

### 4.1. RMS Noise and Magnetic Moment Sensitivity of TMR Sensors

The SQUID microscope has a magnetic moment sensitivity of 10^−15^–10^−14^ Am^2^ at 100 μm [11]. In accordance with the procedure [23], the magnetic-moment sensitivity was estimated from the RMS noise of the TMR sensor. The magnetic dipole moment sensitivity is given as

*m*_min_*=* 4 *×* 10^7^
*h*^3^
*B*_noise_, (1)

where *h* is the minimum lift-off achievable with the instrument, and *B*_noise_ is the measured RMS value of the equivalent magnetic field noise [14]. The RMS in the region outside the sample boundaries is 27 nT for the raw measurements, as shown in Figure 3c (Sensor #3; 178-μm-long TMR sensor), which was used as *B*_noise_. Therefore, for *h* values of 300 μm and 100 μm, the magnetic moment sensitivities were 2.9 × 10^−11^ Am^2^ and 1.1 × 10^−12^ Am^2^, respectively.

The RMS was 11 nT for the TMR Sensor #1 (1073-μm-long). For *h* values of 300 μm and 100 μm, the magnetic moment sensitivities for TMR Sensor #1 (1073-μm-long) were obtained as 1.1 × 10^−11^ Am^2^ and 4 × 10^−13^ Am^2^, respectively. The magnetic moment sensitivity of TMR Sensor #3 was reduced by a factor of three compared with that of TMR Sensor #1. The RMS in the region outside the magnetic point source was 7.6 nT in Figure 4i for the measurements after averaging two images and median filtering of 3 × 3 for the TMR Sensor #3, which leads to the magnetic moment sensitivity of 8.2 × 10^−12^ Am^2^ and 3.1 × 10^−13^ Am^2^ for *h* values of 300 μm and 100 μm, respectively. This is comparable to the magnetic moment sensitivity of the magnetic image measured with TMR Sensor #1 without averaging or a median filter. Thus, the RMS noise can be effectively reduced by averaging multiple magnetic images and median filtering, which eventually leads to an improvement in the magnetic moment sensitivity. A possible future improvement for noise reduction would be to reduce the 1/f noise using measurements at higher frequency intervals. For comparison, we summarize the key statistics for TMR Sensors #1, #2 and #3 in Table 1.

### 4.2. Evaluation of Basalt Magnetic Images Using Upward Continuation

**Figure 5:**
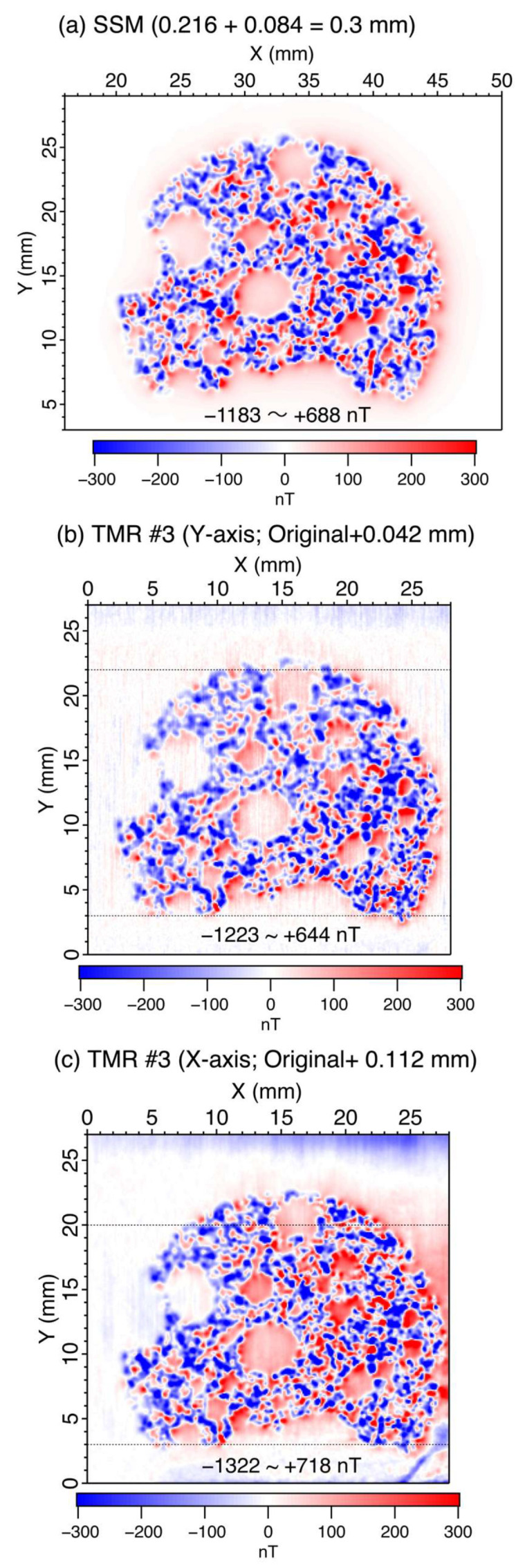
Magnetic images of a basalt thin section after upward continuation. (**a**) Magnetic image measured with SSM. The lift-off was estimated to be 216 μm based on the precision line current, which is upward continued by 84 μm, resulting in an estimated total height of 300 μm. (**b**) Magnetic image measured using TMR sensor #3 with the length direction aligned along the Y-axis. Upward continuation of 42 μm was applied. (**c**) Magnetic image measured using TMR sensor #3 with the length direction aligned along the X-axis. Upward continuation of 112 μm was applied. Magnetic images use a color scale from −300 nT (blue) to +300 nT (red). Values below −300 nT and above −300 nT are clipped to the corresponding end colors. The actual minimum and maximum magnetic field values are indicated in each subfigure. Figure 5a is reproduced from Ref. [23], Figure 4d. The horizontal dotted lines mark the boundaries of the regions used for drift correction, with the outer portions of the image employed for determining the drift.

To compare the magnetic-field images obtained for TMR Sensor #3 in the two longitudinal directions, an upward continuation was applied. First, an upward continuation of 84 μm was applied to the SSM magnetic image to achieve a total lift-off of 300 μm (Figure 5a). The minimum and maximum magnetic field values are −1193 nT and 688 nT, respectively. For TMR Sensor #3 on the Y-axis, an upward continuation of 42 μm was applied to achieve magnetic field values similar to those of the SSM magnetic image shown in Figure 5a,b. The resulting minimum and maximum magnetic field values are −1223 nT and 644 nT, respectively. Similarly, an upward continuation of 112 μm was applied to TMR Sensor #3 in the X-axis, with minimum and maximum magnetic field values of −1322 nT and 718 nT, respectively. (Figure 5c). Notably, the patterns in the magnetic images in Figure 5b,c were almost identical, except for a positive background in Figure 5c with slightly higher values. Although the spatial resolution of the SSM magnetic image was similar to that of the two magnetic images for TMR Sensor #3 (Figure 5b,c), the details of the patterns were different. We suspect that the differences are caused either by an integration over the vertical length of a free layer (100 μm) in the TMR sensor or by an interaction between the magnetic field leaking from the TMR sensor and the basalt sample.

**Figure 6:**
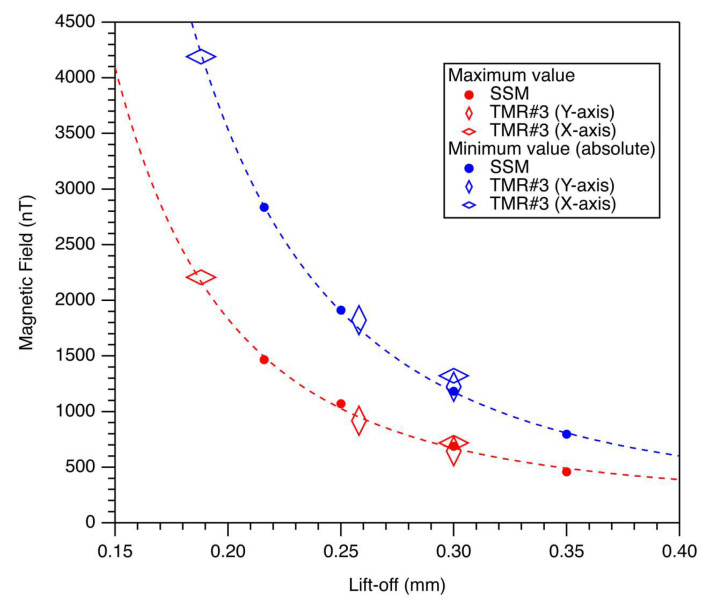
Magnetic field values for magnetic images before and after upward continuations. Red and blue circles represent the absolute values of the maximum and minimum magnetic fields for the basalt thin section measured using the SSM with a lift-off of 212 μm, together with those after upward continuations to 250 μm, 300 μm, and 350 μm, respectively. Red and blue dashed lines are theoretical curves inversely proportional to the cube of the lift-offs for maximum and minimum field values, assuming a dipolar magnetic field, respectively. Red (blue) vertically elongated diamonds indicate the maximum (minimum) field values in the magnetic image measured with the TMR sensor, with the longitudinal direction aligned along the Y-axis. A lift-off of 258 μm is assumed to be consistent with the dashed curves, and an upward continuation of 42 μm (total virtual lift-off of 300 μm) was applied. Red (blue) horizontally elongated diamonds represent the maximum (minimum) field values in the magnetic image measured with the TMR sensor, with the longitudinal direction aligned along the X-axis. A lift-off of 188 μm is assumed to be consistent with the dashed curves, and an upward continuation of 112 μm (total virtual lift-off of 300 μm) was applied.

To estimate and evaluate the lift-off values before and after the upward continuation of the TMR sensor, comparisons were made with SSM magnetic images before and after the upward continuation (Figure 6). Magnetic images for the SSM measured with a lift-off value of 216 μm (Figure 3b) were upward continued to 250 μm (Figure 5a), 300 μm, and 350 μm. The minimum (absolute) and maximum values were plotted against the expected total liftoff values. Theoretical curves inversely proportional to the cube of the lift-offs were drawn, assuming a power law for a dipolar magnetic field (blue and red dashed lines in Figure 6). Further, minimum and maximum values were plotted versus the expected lift-off values before and after upward continuation for TMR Sensor #3 in the Y-axis, starting from the assumed original lift-off value of 258 μm to the total lift-off value of 300 μm (blue and red vertically elongated diamonds). Similarly, the minimum and maximum values were plotted versus the expected lift-off values before and after upward continuation for TMR Sensor #3 in the X-axis, starting from the assumed original lift-off value of 188 μm to the total lift-off value of 300 μm (blue and red horizontally elongated diamonds). Notably, the minimum and maximum magnetic field values for TMR Sensor #3 with length alignments in both directions were consistent with the curve fitted to the cube of the lift-offs, assuming dipolar magnetic fields. This confirms the lift-off estimates for the magnetic field measurements using TMR Sensor #3, with length alignments along the Y- (258 μm) and X-axes (188 μm), based on comparisons of the minimum and maximum magnetic field values in the SSM image.

### 4.3. Characterization of TMR Sensors Using Point-Source Magnetic Images

The full width at half maximum (FWHM) of the peak is an effective parameter for evaluating the spatial resolution of magnetic sensors. It also allows us to evaluate the distortions caused by the elongated shape of the sensitivity region. Figure 4d,h,l show the FWHM values measured along the X-(red circles) and Y-axes (orange circles). The FWHM for the magnetic image measured for the point source with the SSM was 240 μm, the same for the X- and Y-axes. This is consistent with the square shape of the SQUID washer (200 μm × 200 μm) and hole (30 μm × 30 μm) [11,12,25]. The FWHM for the magnetic image measured using TMR Sensor #3, with the length along the X-axis, was 200 μm in the X-axis and 210 μm in the Y-axis. In contrast, the FWHM for the magnetic image measured using TMR Sensor #3 with the length along the Y-axis was 170 μm and 160 μm for X- and Y-axes, respectively. In addition to the visual inspections on the basalt magnetic images shown in Section 3.2, these results confirm that the distortions are caused by the anisotropic shape of the TMR sensor. Hence, the distortions of the sensing region are negligible and do not affect the symmetry of the spatial resolution along the X- and Y-axes at distances of 200 μm and 170 μm, respectively, which are sufficiently large compared with the TMR sensor length.

**Figure 7:**
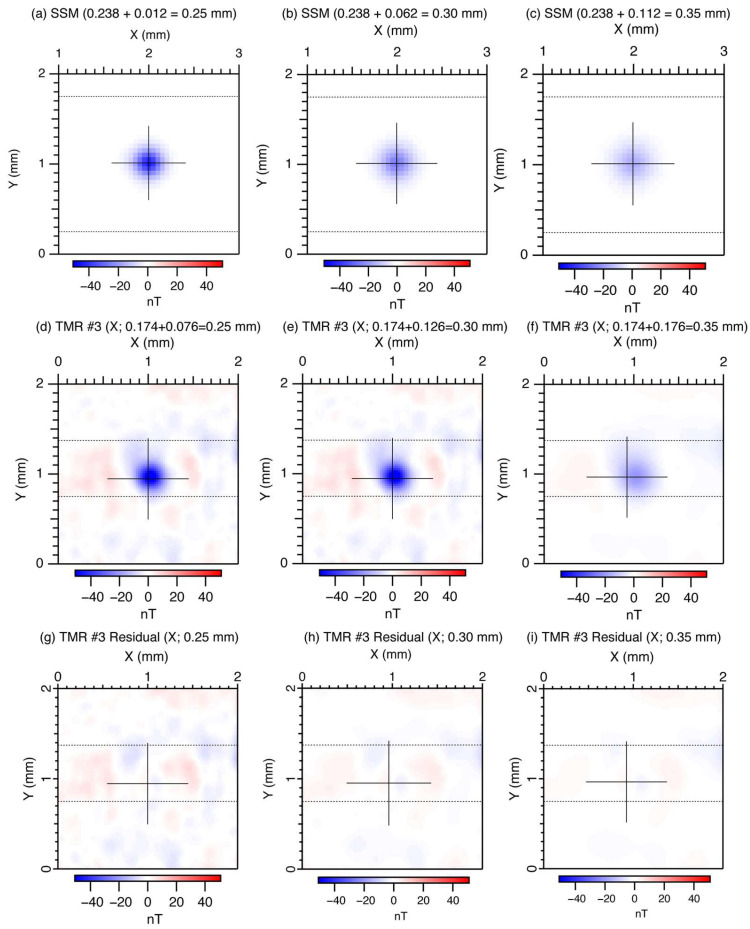
Magnetic images of the point source after upward continuation. (**a**–**c**) Magnetic images measured with the SSM after upward continuations of 12 μm, 62 μm, and 112 μm, respectively, from the original lift-off of 238 μm. (**d**–**f**) Magnetic images measured with TMR Sensor #3 with the longitudinal direction aligned along X-axis after upward continuations of 76 μm, 126 μm, and 176 μm, respectively, from the original lift-off of 174 μm. (**g**–**i**) Residual magnetic field images after subtraction of a best-fit dipole for the corresponding magnetic images. The thin black cross in each image indicates the position of the best-fit dipole. The horizontal dotted lines mark the boundaries of the regions used for drift correction, with the outer portions of the image employed for determining the drift.

Figure 7 shows the magnetic images of the point source with vertically downward magnetization (50 μm × 50 μm square) after upward continuation. Figure 7a–c show the upward continued magnetic images to total lift-off values of 250 μm, 300 μm, and 350 μm, originally measured using the SSM with an estimated lift-off of 238 μm as shown in Figure 4b. It should be noted that the cross marks indicating the positions of the fitted dipoles are centered at the minimum peak value. Figure 7d–f are the upward-continued magnetic images for total lift-off of 250 μm, 300 μm, and 350 μm, originally measured using TMR Sensor #3, with an estimated lift-off of 174 μm, as shown in Figure 4j. It should be noted that the cross marks indicating the positions of the fitted dipoles are slightly positioned away from the minimum peak value, which might be related to an inclined dipole due to the positive magnetic field values located around the left side of the dipole. Figure 7g–i are the residual magnetic fields after subtraction of the fitted dipole from the measured magnetic images after upward continuation, as shown in Figure 7d–f.

**Figure 8:**
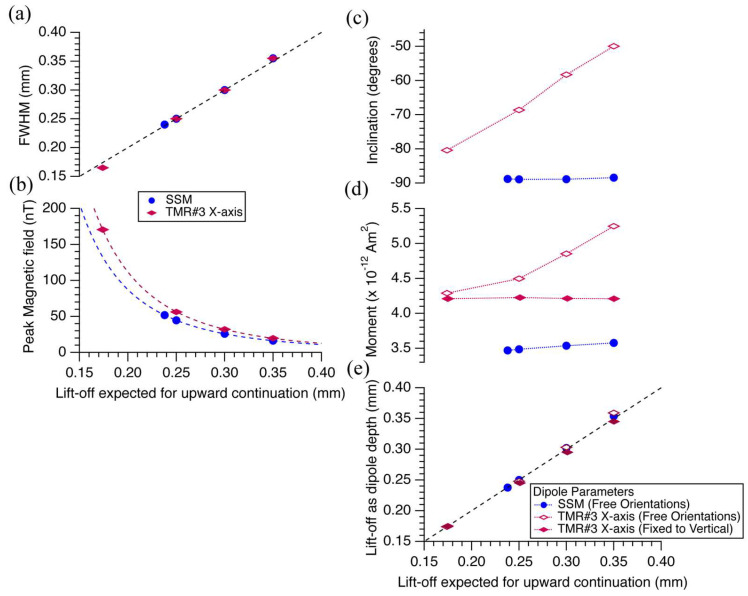
Parameters of magnetic images and dipole fitting for the magnetic point source before and after upward continuation at total distances of 250 μm, 300 μm, and 350 μm. The lift-offs for the SSM and TMR Sensor #3 (X-axis) before upward continuation are estimated to be 238 μm and 174 μm, respectively, based on the dipole fit. (**a**) Full width half maximum, and (**b**) peak magnetic field (negative values) for the SSM (blue circle) and the TMR (purple diamonds) magnetic images. Blue and purple dashed lines represent theoretical curves inversely proportional to the cube of the lift-offs for the magnetic field values, assuming a dipolar magnetic field, respectively. (**c**) Inclination, (**d**) magnetic moment, and (**e**) lift-off values calculated as depths for the best-fit dipoles for the SSM (blue circle) and the TMR (purple diamonds) magnetic images. Open diamonds are the parameters for dipoles of free orientation without constraint. In contrast, solid diamonds are those for vertical dipoles with a fixed inclination of −90° (or zero horizontal magnetic moment values). Solid black dashed lines in Figure 8a,e are lines with slope 1.

Figure 8 summarizes the magnetic field image parameters for the magnetic point source and the fittings of the dipole in Figure 4 and Figure 7. Figure 8a shows the FWHM for the point source measured using the TMR sensor along the X-axis (purple diamonds) and that for the SSM (blue circles). Both are positioned on a line with slope 1, suggesting that the spatial resolutions (FWHMs) of the magnetic point source for the SSM and TMR sensors are approximately equal to the lift-off values. Figure 8b shows the peak magnetic field (absolute value of the minimum values) for the SSM (blue circles) and TMR sensors (purple diamonds). The theoretical curves fit well to the magnetic field values, which are inversely proportional to the cube of the lift-off, assuming the power-law behavior of a dipolar magnetic field.

Dipole fit on the SSM magnetic image before and after upward continuation yields inclination of approximately −89° (blue circles; Figure 8c). The magnetic moment values are approximately 3.5 × 10^−12^ Am^2^ with a slight increase with increasing lift-off values (blue circles; Figure 8d). The lift-off values, calculated as the dipole depths, lie on a line with slope 1 (blue circles; Figure 8e). In contrast, the dipole fit on the TMR magnetic image yields inclination values starting at approximately −80° before upward continuation, which increases to approximately −50° at a lift-off value of 350 μm (open purple diamonds; Figure 8c). Furthermore, the magnetic moment is approximately 4.3 × 10^−12^ Am^2^ before upward continuation, increasing to approximately 4.3 × 10^−12^ Am^2^ at a lift-off of 350 μm. The liftoff values calculated as the dipole depth are close to but slightly above the line with slope 1 (open purple diamonds; Figure 8d).

For evaluation, we assumed a vertical dipole with zero horizontal magnetic moment and applied this model to each magnetic field map. Stepwise upward continuation to 0.25, 0.30, and 0.35 mm yielded virtually identical dipole-fit magnetic moments of approximately 4.3 × 10^−12^ Am^2^ at all lift-off levels (solid purple diamonds; Figure 8c). This indicates that constraining the inclination to −90° suppresses the magnetic-moment estimates to physically reasonable values. In addition, the lift-off values obtained as effective dipole depths from the vertical-dipole fits (solid purple diamonds) lie closer to the 1:1 line (black dashed line) than those obtained without the vertical constraint (open purple diamonds) in Figure 8d, indicating that the corresponding depth estimates are also improved by the vertical constraint. Using SSM estimates as a benchmark, the deviation from physically reasonable values observed in the unconstrained dipole fits likely arises from noise or long-term drift in the magnetic field images. The magnetic moment values can be reduced to values closer to those of the SSM by introducing a vertical-dipole constraint, which supports the above interpretation. However, there are inconsistencies in the peak magnetic field values (Figure 8b) and magnetic moment estimates (Figure 8d) for the SSM and TMR sensors. The discrepancy could be due to an integration over the vertical length of a free layer (100 μm) in the TMR sensor, which differs from the observation with a flat superconducting pickup coil in the SSM. Future improvements could aim to reduce these discrepancies.

## 5. Conclusions

In this study, a 178 μm long TMR sensor with a pair of TMR devices was prepared with a sensitivity of 700 nT/V. Sensor performance was evaluated using a geological thin section and a point source and compared with the SSM. The PSD for the TMR sensor was approximately 60 nT/√Hz at 10 Hz. The RMS noise of the background during a typical scan operation was 27 nT for the TMR sensor yielding magnetic moment sensitivities of 2.9 × 10^−11^ Am^2^ and 1.1 × 10^−12^ Am^2^ for lift-off values of 300 μm and 100 μm, respectively. By averaging two images and applying a 3 × 3 median filter, the RMS noise was reduced to 7.6 nT, leading to magnetic moment sensitivities of 8.2 × 10^−11^ Am^2^ and 3.0 × 10^−13^ Am^2^, respectively.

Magnetic images were obtained for a thin geological section of Hawaiian basalt with vertical magnetization. The measurements were performed in two configurations, with lengths parallel to the X- (lift-off = 174 μm) and Y-axes (lift-off = 200 μm), showing no elongation due to the asymmetry of the sensing region. Although the two magnetic images were nearly identical after upward continuation to a distance of 300 μm, the detailed patterns differed from those in the SSM image. These differences could be caused either by a vertically elongated sensing region (100 μm) in the TMR sensor or by the magnetic field generated by the TMR sensor.

For the evaluation of the TMR sensor, a magnetic point source (50 μm × 50 μm square) with perpendicular magnetization was prepared. The film consisted of a thin CoFe layer sandwiched between two MgO layers. The magnetic image measured with the SSM showed a clean image of a magnetic dipole with a tilt angle of approximately 1° from the vertical direction. The point source was measured using a TMR sensor that could be fitted to a magnetic dipole. The FWHM values for both the SSM and TMR sensors closely matched the liftoff values, with no asymmetry. However, the peak magnetic fields, magnetic moments, and the dipole tilts for the TMR sensor were significantly larger than those of the SSM, which may imply the discrepancy caused either by the vertical length of a sensing region (100 μm) of the TMR sensor or by a magnetic field leaking from the TMR sensor.

## Figures and Tables

**Table 1:** Comparison of TMR sensors for scanning magnetic microscopy, adapted from MEG sensor designs.

TMR Sensor	Dimension (μm)			PSD@1Hz ^1^ (nT/√Hz)	RMS noise ^2^ (nT)	Number of Images & Filters	Magnetic Moment Sensitivity (Am^2^)		Literature
	Length	Width	Height				Lift-Off 300 μm	Lift-Off 100 μm	
TMR #1	1073	0.1	100	~200	11	1 image No filter	1.1 × 10^−11^	4.0 × 10^−13^	[23]
TMR #2	357	0.1	100	~600	-	-	-	-	[23]
TMR #3	178	0.1	100	~900	27	1 image No filter	2.9 × 10^−11^	1.1 × 10^−12^	This study
TMR #3	178	0.1	100	~900	7.6	2 images median filter	8.2 × 10^−12^	3.1 × 10^−13^	This study

^1^ Power Spectral Density (PSD) estimated from Figure 2b. ^2^ RMS noise estimated from measurements on the sample-free glass area of the basalt thin-section slide.

**Figure A1:**
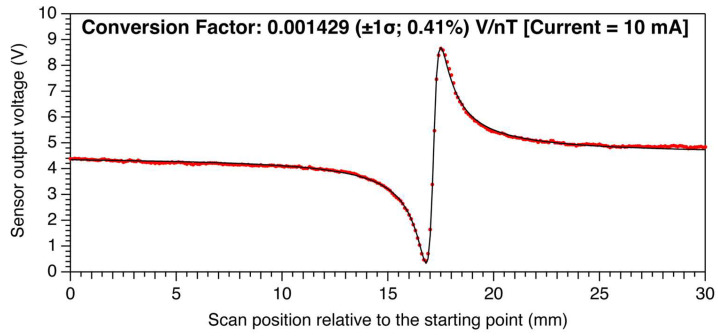
Sensor output voltage measured with the TMR sensor (Sensor #3) while scanning across a straight line current of 10 mA supplied by a precision current source (red dots). The black line shows the best-fit infinite straight-wire model. Fitting to the line-current model was performed using SQUIDLineScan Version 2.02b [11].

## Data Availability

The datasets analyzed in this study are publicly available on Zenodo at https://zenodo.org/records/18057439 (doi:10.5281/zenodo.18057439, accessed on 26 December 2025).

## Abbreviations

The following abbreviations are used in this manuscript:

SMM	Scanning magnetic microscopy
SQUID	Superconducting quantum interference device
TMR	Tunnel magneto-resistance
ARM	Anhysteretic remanent magnetization
MEG	Magnetoencephalography
MTJ	Magnetic tunnel junction
SSM	Scanning SQUID microscope
AIST	National Institute of Advanced Industrial Science and Technology
GSJ	Geological Survey of Japan
FWHM	Full width at half maximum
PSD	Power spectral density
FFT	Fast Fourier transform

## Appendix A

The calibration for the sensitivity of the TMR sensor was performed by applying a line current from a precision current source (Model 121, Lake Shore Cryotronics, Westerville, OH, USA) through a straight aluminum wire of 25-μm diameter on a glass plate coated with a thin film [11]. The sensitivity calibration of the TMR sensor was performed according to the methodology described in our previous study [23]. Measurements were taken at intervals of 100 μm along a line on the Y-axis with a line current of 10 mA. To maximize the uniformity of the magnetic field within the sensing region, the longitudinal direction of the TMR sensor was aligned parallel to the line current. Using the SQUIDLineScan software Version 2.02b [11], the measured sensor output voltage data were fitted to the theoretical curve for an infinite straight-line current (Figure A1). The apparent conversion factor, which is expected to be unity for a correctly configured sensitivity setting, was determined to be 0.001429 (1σ; 0.41%) for a preset value of unity. Using this conversion factor, the calibration factor, defined as the magnetic field per unit sensor output voltage, was determined to be 700 nT/V (=1/0.001429). This value was applied to the magnetic field calculations throughout this study.
